# Biofilm and Diatom Succession on Polyethylene (PE) and Biodegradable Plastic Bags in Two Marine Habitats: Early Signs of Degradation in the Pelagic and Benthic Zone?

**DOI:** 10.1371/journal.pone.0137201

**Published:** 2015-09-22

**Authors:** Andreas Eich, Tobias Mildenberger, Christian Laforsch, Miriam Weber

**Affiliations:** 1 Biology/Chemistry Department, University of Bremen, Bibliothekstraße 1, 28359 Bremen, Germany; 2 Animal Ecology I, University of Bayreuth and BayCEER, Universitaetsstraße 30, 95447 Bayreuth, Germany; 3 HYDRA Institute for Marine Sciences, Elba Field Station, 57034 Campo nell’Elba, Italy; University of Sydney, AUSTRALIA

## Abstract

The production of biodegradable plastic is increasing. Given the augmented littering of these products an increasing input into the sea is expected. Previous laboratory experiments have shown that degradation of plastic starts within days to weeks. Little is known about the early composition and activity of biofilms found on biodegradable and conventional plastic debris and its correlation to degradation in the marine environment. In this study we investigated the early formation of biofilms on plastic shopper bags and its consequences for the degradation of plastic. Samples of polyethylene and biodegradable plastic were tested in the Mediterranean Sea for 15 and 33 days. The samples were distributed equally to a shallow benthic (sedimentary seafloor at 6 m water depth) and a pelagic habitat (3 m water depth) to compare the impact of these different environments on fouling and degradation. The amount of biofilm increased on both plastic types and in both habitats. The diatom abundance and diversity differed significantly between the habitats and the plastic types. Diatoms were more abundant on samples from the pelagic zone. We anticipate that specific surface properties of the polymer types induced different biofilm communities on both plastic types. Additionally, different environmental conditions between the benthic and pelagic experimental site such as light intensity and shear forces may have influenced unequal colonisation between these habitats. The oxygen production rate was negative for all samples, indicating that the initial biofilm on marine plastic litter consumes oxygen, regardless of the plastic type or if exposed in the pelagic or the benthic zone. Mechanical tests did not reveal degradation within one month of exposure. However, scanning electron microscopy (SEM) analysis displayed potential signs of degradation on the plastic surface, which differed between both plastic types. This study indicates that the early biofilm formation and composition are affected by the plastic type and habitat. Further, it reveals that already within two weeks biodegradable plastic shows signs of degradation in the benthic and pelagic habitat.

## Introduction

The production of plastics has increased over the past 50 years. In 2012, 288 million tons of plastics were produced worldwide [1]. Most of it is fabricated for single use only, like packaging materials, which represented around 40% of the European plastic demand in 2012 [1]. After disposal, plastic litter can be introduced into the marine environment by rivers, airborne transport, storm events, and tsunamis [2–5]. One of the largest contributors to marine plastic litter are discarded products from recreational and commercial seafaring [6–9]. Related environmental consequences include the entanglement of animals, mechanical impairments of swallowed plastics mistaken as food, accumulation of persistent organic pollutants (POPs) and the transportation of harmful algae and invasive species [8,10,11]. Newest modelling data predict that the input to the sea will increase in the order of one magnitude until 2025, if no changes in the waste management occur [12].

Plastic is considered stable with an estimated degradation time of several hundred years depending on the plastic type [8]. Many plastic types are considered to be bio-inert [6] and thus not biodegradable [13], due to hydrophobicity and high molecular weight of synthetic polymers which prevents phagocytosis [14]. Consequently, microbial plastic decomposition is estimated to be generally low, even though some microorganisms are capable of degrading plastic [15].

Besides efforts to reduce plastic usage and enhance plastic recycling, performing research on biodegradable plastics is strongly encouraged by organisations like UNEP and NOAA [16]. Italy banned conventional plastic shopping bags in 2011 [17], and biodegradable plastic bags have taken their place. Since the European parliament has voted to limit the use of plastic bags and reduce pollution other countries will follow the example of Italy, which may result in a higher input of biodegradable plastic into the marine environment. The production of biodegradable plastic is predicted to increase from 604,000 tons in 2012 to over 1,000,000 tons in 2017 [18]. Degradable plastic is defined as plastic “in which the degradation results from the action of naturally-occurring microorganisms such as bacteria, fungi and algae” [19]. Examples for these materials are starch composites, polyvinyl alcohol, polycaprolactone, poly(hydroxyl butyrate valerate) and polylactates [20]. O’Brine and Thompson [21] observed differences in the time of degradation for different polymer types introduced into the marine environment for 40 weeks. One type of biodegradable plastic had disappeared completely, while other biodegradable plastics and conventional high-density polyethylene (HDPE) showed only few changes of the surface area within the same time frame.

Any surface exposed to seawater is colonised by organisms [22], starting with microorganisms which form a biofilm. Organisms growing on the plastic may therefore influence degradation. Diatoms are among the first colonisers of surfaces in the sea and are found in great densities, playing an important role in biofilm formation [23,24] and the overall biogeochemical activity within the biofilm. In experiments in the English Channel, Cunliffe et al. [25] detected a biofilm on polyethylene (PE) sample surfaces after one week, which kept on growing for the following two weeks. During longer experiments of 6 and 12 months performed in the Bay of Bengal, India, the biofilm amount varied over time [22,26]. The authors suggested that seasonal influences, such as nutrient availability, light intensity or temperature changes might have caused these variations. With growing biofilm, invertebrates are attracted [27] and a community of photo- and heterotrophic organisms establishes. So far, both the composition of the biofilm community and its activity in relation to environmental settings and plastic degradation remains to be investigated in more detail.

Fouling decreases the buoyancy of plastic leading to sinking of previously floating plastic [28]. Around 70% off all plastic particles in the sea will eventually sink to the seafloor [29]. In the pelagic and benthic zone plastic debris faces different environmental forces. For example, more light will reach a floating plastic bag near the water surface than a plastic bag sunken to the benthic zone. In contrast, at the sedimentary seafloor shear forces are high when waves drag the plastic item over the sediment. Until now most studies have focussed on floating plastic particles. The fouling and degradation processes of sunken plastic debris have not yet been studied sufficiently.

In this pilot study we investigated the effects on plastic bags that were exposed to the marine environment for one month. Since few studies have been performed focussing on the early stages of biofilm succession and activity and since laboratory tests have revealed that early signs of degradation occur within a few weeks [30], we aimed to test if this holds true when plastic is exposed to field conditions. Biological and physical parameters were measured and compared between conventional PE shopper bags and bags made of biodegradable plastic exposed to a benthic and pelagic habitat. We assessed biofilm formation particularly focussing on the abundance and composition of diatoms. We measured oxygen production to assess whether there is production or depletion of oxygen caused by the biofilm or the polymers themselves. The degradation processes were studied in terms of tensile properties by use of a dynamometer, and by changes in the polymer surface, which was visualised by scanning SEM.

## Material and Methods

The study required no permit or approval as there we no ethical aspects raised. The field tests took place in a public marine area without any protection status, there were no animals involved, nor any environmental items sampled. The field tests consisted of a short-term exposure of non-hazardous artificial materials (concrete bricks, rope, plastic) in a marine coastal environment characterized by physical mixing and sediment redistribution (sand bottom). All materials were retrieved completely after the tests, posing no source of pollution or impact to the marine environment.

In this study we tested two types of plastic: conventional polyethylene and the biodegradable plastic Mater-Bi (N°014). Both types of plastic are available in form of carrier bags and were obtained from local supermarkets. Mater-Bi N°014 is a starch-based biopolymer–polyethylene terephthalate blend, that consists of renewable monomers made from vegetable oils and corn starch. It meets the compostability requirements of the EN 13432 [30,31].

The experiment was carried out in the Mediterranean Sea at the Bay of Fetovaia, Elba, Italy (42° 43' 49.01" N, 10° 9' 20.88" E). During the exposure time, the average water temperature at both experimental sites was 14.9 ± 1.0°C (standard error). In total, 320 plastic samples (15.5 x 5.4 cm) were attached to 30 PVC plates and deployed at two sites representing the benthic and the pelagic zone: directly above the sediment surface in 6 m water depth, simulating a plastic bag sunken to the seafloor and in the water column in 3 m depth, simulating a plastic bag floating in the water column.

Starting in April 2013, the plastic samples were exposed for 15 and 33 days. After retrieving, the samples were gently plunged into sterile seawater (Millipore, ‘Durapore’ 0.22 μm) and cut in subsamples. For each method the exact area of the samples—photographed using a desk scanner (Canon, ‘LiDE 60’)—was measured by means of the software ImageJ [32]. Obtained values were standardised to a sample surface of 1 cm^2^.

### Biofilm amount

A quantitative biofilm assay after Cunliffe et al. [25] was performed with the following modifications. Samples (1 x 4 cm, n = 10) were air-dried for 45 min. A total of three drops of crystal violet (Sigma, 1% aqueous solution) were applied to the samples. Then the samples were rinsed with sterile seawater until no stain was visible. After 45 min of air-drying the stain trapped in the biofilm was extracted for 10 min in 1.2 mL ethanol (AppliChem, 96% p.A.). The extinction of the samples was measured using a photometer (HACH, ‘DREL 2400’) at a wavelength of 595 nm (blank: ethanol).

### Diatom abundance and composition

**Fig 1:**
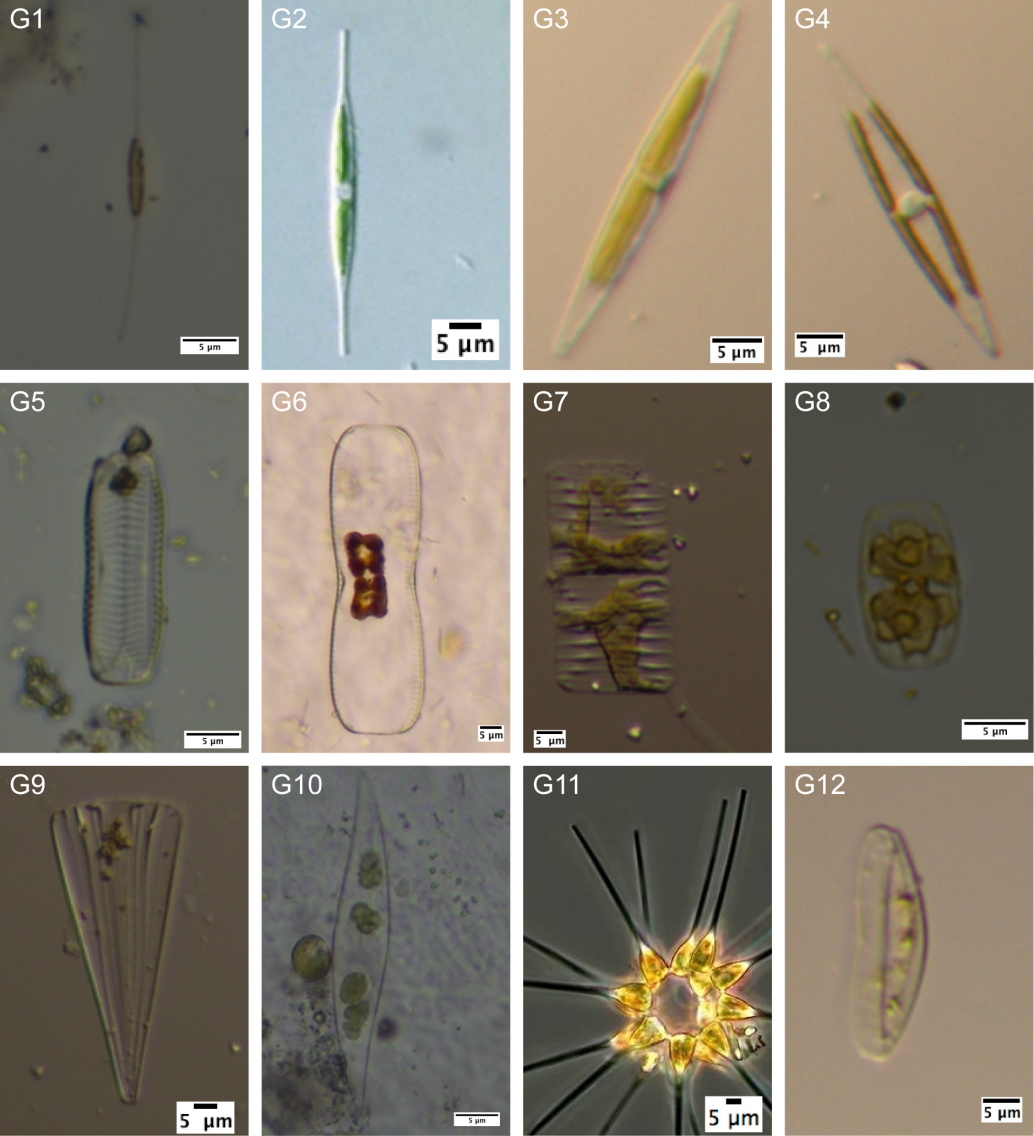
Photo of representatives for each diatom group based on morphological features. No example is shown for group G13 (i.e. unknown diatoms) in this figure. Image source for G2: [33] modified, and for G11: [34] modified.

Diatoms were chosen as the model organisms for benthic microalgae within a biofilm. They are a major component of marine biofilms and play an important role during their formation and activity. Furthermore, diatoms are organisms that impact the oxygen production patterns of the plastic fouling community and of the surrounding ecosystem. According to Patil and Anil [23] the biofilm was removed from the samples (10 x 4 cm, n = 6) with a toothbrush and 30 mL sterile seawater. The suspension was transferred from a bowl into a 50 mL Sarstaedt tube. Subsequently, the bowl was rinsed with 20 mL sterile seawater (SSW), which was added to the tube afterwards. Seven drops of Lugol’s iodine (MERCK, 5%) were used to preserve the organisms. After a sedimentation time of two days 45 mL supernatant was removed. The remaining 5 mL was mixed and a subsample of 1 mL was transferred into a Sedgewick-Rafter counting chamber (Physer-SGI, Sedgewick Rafter Cell S53) for microscopical analysis (400x magnification, Zeiss, Axiostar plus). Diatoms within a size range of 10–200 μm were considered. The diatoms were counted and classified into 13 morphological groups from G1 to G13, with G13 comprising all unclassifiable diatoms (Table 1). Fig 1 shows a representative for each morphological group [33, 34]. The analysis was performed until either 75 fields were analysed or a total number of 75 organisms were detected.

**Table 1:** Diatom groups based on morphological features.

Group	Morphology	Genus
G1	frustule with long, narrow ends	*Cylindrotheca* spp.?
G2	frustule with shorter ends than G1, chloroplasts apical	*Nitzschia* spp.?
G3	rounded/angled or pointed-ellipsoidal frustule, chloroplasts apical	*Nitzschia* spp.?
G4	rounded/angled or pointed-ellipsoidal frustule, chloroplasts lateral	*Navicula* spp.?
G5	rounded frustule	*Amphora* spp.?
G6	rounded frustule, axial constriction	*Diploneis* ssp.? *Amphora* ssp.?
G7	angled frustule, star shaped chloroplast, often stalked	*Striatella* spp.?
G8	rounded frustule, rounded caps at the ends	*Amphora* spp.?
G9	triangular frustule, araphide diatom	*Licmophora* spp.?
G10	sigmoide frustule	*Pleurosigma* spp.?, *Gyrosigma* spp.?
G11	frustule with a thickened, triangular (in valvar view rounded) apex	*Asterionellopsis* spp.?
G12	crescent frustule	
G13	Not known	

### Oxygen production

SCHOTT bottles (100 mL) with samples (10 x 4 cm, n = 10) incubated in sterile seawater were exposed to light (Osram, BIOLUX L 36W/965, light intensity: 1577 ± 38 lux (standard error)) for about 8 h. Subsequently, the samples were kept in darkness for about 12 h. Before and after each incubation the oxygen concentration of the sterile seawater was measured with clark type microsensors [35]. On the basis of the changes in the oxygen concentration, the total oxygen production rate per day and plastic surface were calculated assuming that a day consists of 12 h of darkness and 12 h of light.

### Tensile properties

The tensile strength was measured using a dynamometer (Dinamometro INSTRON 5500), which pulls inserted plastic samples (10 x 1 cm, n = 4) until the specimens break. The results of this measurement are given in: maximal elongation at break (epsilon) and force needed to break (sigma).

### SEM analysis

To get an insight into the small-scale changes of the polymer surface, samples were analysed using a scanning electron microscope (SEM) (FESEM, LEO 1530 VP, LEO Elektronenmikroskopie GmbH, Oberkochen, Germany, magnification 100x—10,000x, 3 kV, aperture size: 30 μm standard, SE2 detector). The samples were rinsed with freshwater and air-dried to remove most of the biofilm without damaging the polymer surface. Due to the gentle cleaning, a small amount of remaining biofilm was still visible. Randomly taken subsamples of 5 to 10 mm side length were cut and fixed on aluminium stubs (Plano GmbH Elektronenmikroskopie, Wetzla, Germany) using double coated carbon conductive tabs (Plano GmbH Elektronenmikroskopie, Wetzla, Germany). Samples were subsequently coated with a 1.3 nm thick platinum layer (Cressington HR208 sputter coater and a Cressington mtm20 thickness controller) and analyzed using SEM.

### Statistics

Data represents means ± 1 standard error. All statistical analyses were performed using the statistical software RStudio [36] and the packages vegan [37], car [38] and sciplot [39]. Generalised linear models (GLM) with three-way interactions (plastic type*site*time) were applied and a backwards stepwise model minimization according to the Akaike Information Criteria (AIC) was performed. The models were then assessed by means of ANOVA (F-Tests or Chi square tests). *p* values of <0.05 were used as standard for statistical significance.

The diatom composition was analysed using Detrended Correspondence Analysis (DCA). The results are shown in a scatterplot in which the diatom composition on each plastic sample is represented by a point. Samples with a similar diatom composition are plotted closer to one another than samples with a less similar composition. The samples were grouped by plastic type and sampling time. Around the centroid of all points for these groups an ellipse indicating the 95% confidence interval of their standard error was drawn. If these ellipses did not overlap the diatom composition was significantly different (P ≤ 0.05) in respect of plastic type and sampling time.

## Results

### Biofilm amount

**Fig 2:**
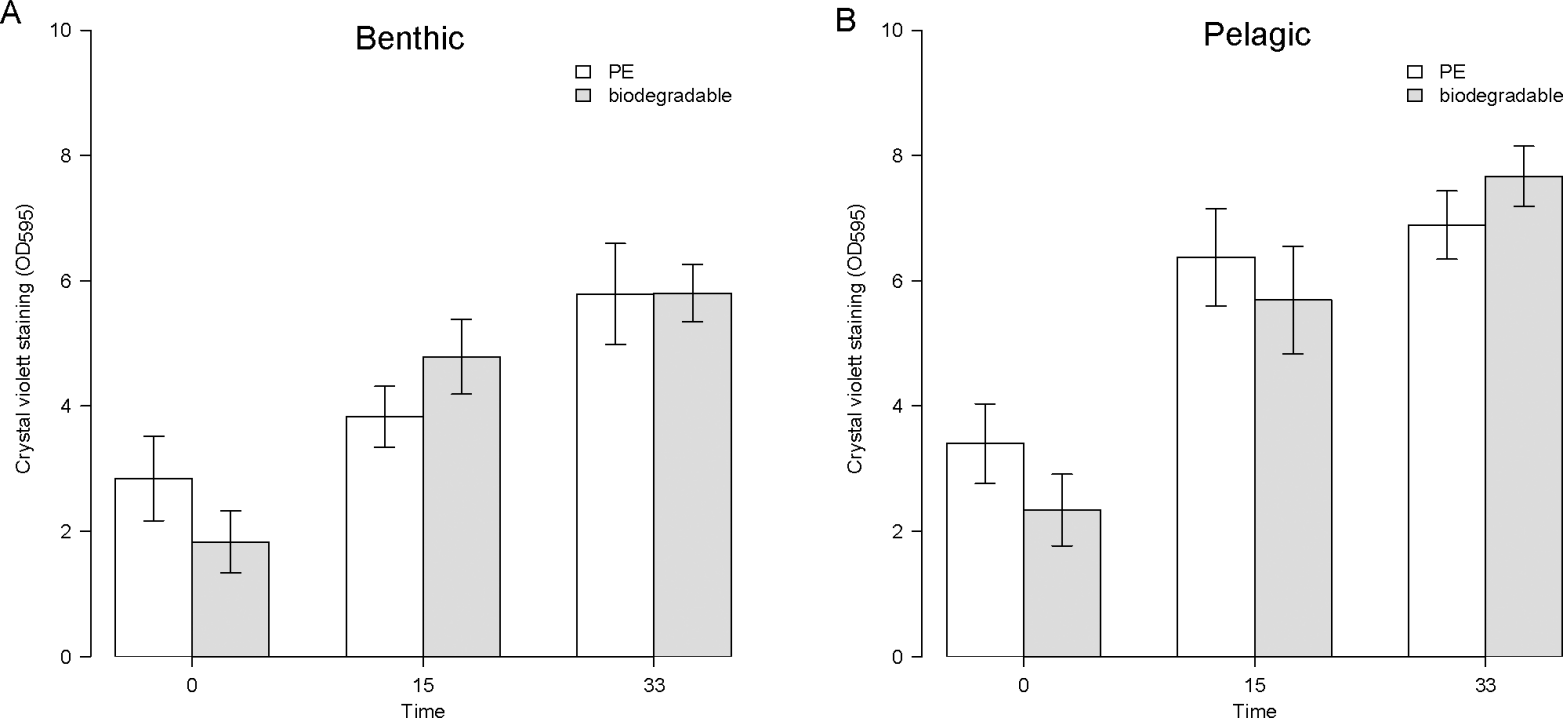
Biofilm development over time. The relative amount of biofilm found on PE (white) and biodegradable plastic (grey) after 15 and after 33 days of the experiment. Time 0 represents the polymer without biofilm. The error bars indicate the standard error. (A) Results of benthic samples (6 m water depth). (B) Results of pelagic samples (3 m water depth).

Within 33 days the amount of biofilm increased significantly (Fig 2) on all plastic samples (time: *Chi* ^2^(2, *N* = 10) = 63.004, *p* < 0.001, Table 2). The value of crystal violet staining (OD595) increased from to 5.17 ± 0.73 after two weeks to 6.54 ± 0.62 after 4 weeks. Results show that neither the site nor the plastic type affected the temporal change in biofilm amount significantly (Table 2).

**Table 2:** Biofilm amount in relation to plastic type, site and exposure time.

	*Chi* ^2^ ^a^	*df* ^b^	*p* ^c^
plastic type	0.000	1	0.989
site	10.696	1	0.001
time	63.004	2	<0.001
plastic type*time	5.424	2	0.066

^a^ likelihood ratio of *Chi* ^2^ value

^b^ degrees of freedom

^c^ probability value

### Diatom abundance and composition

**Fig 3:**
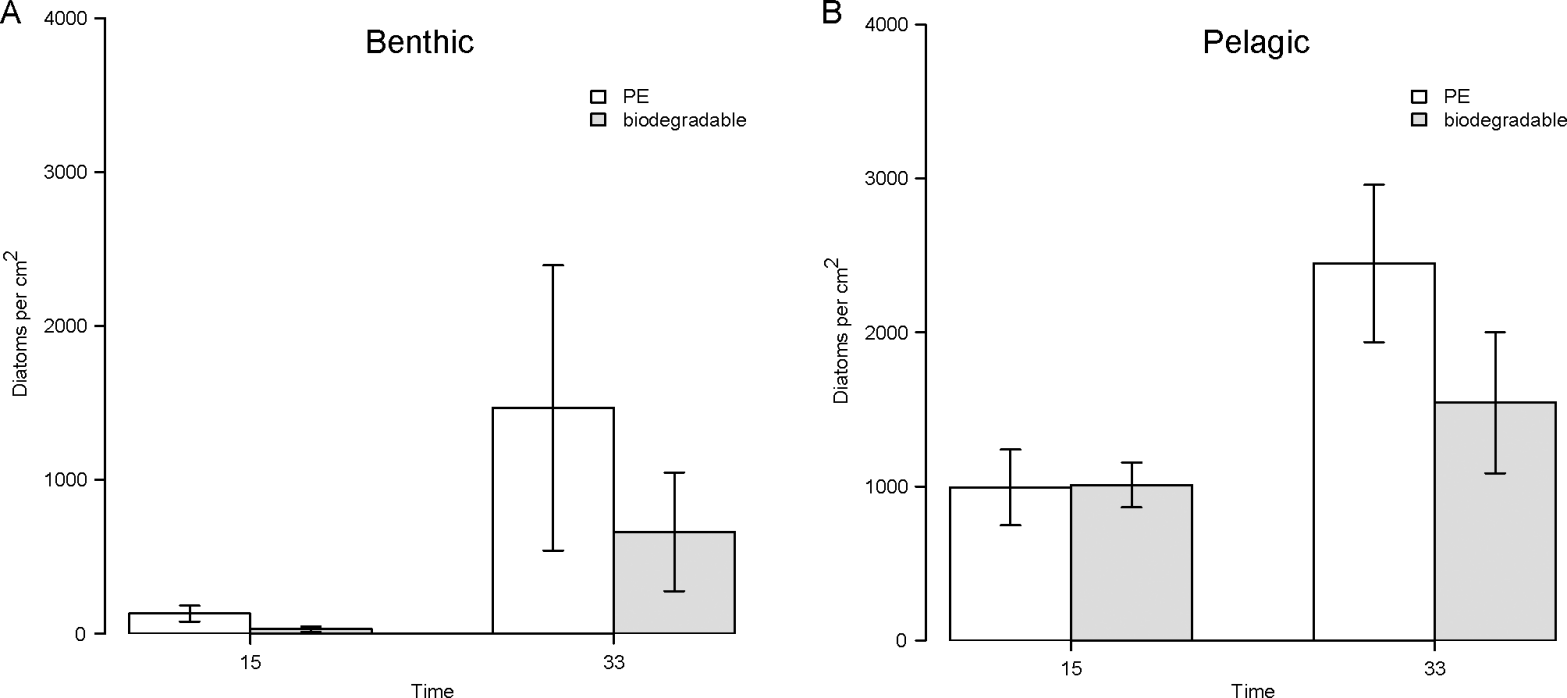
Diatom abundance per treatment. The number of diatoms found per square centimetre on PE (white) and biodegradable plastic (grey) is displayed after 15 and 33 days of the experiments. The error bars indicate the standard error. (A) Results of benthic samples (6 m water depth). (B) Results of pelagic samples (3 m water depth).

The number of diatoms increased significantly on all samples (time: *F*(1,40) = 13.534, *p* < 0.001, Table 3, Fig 3). The habitat affected the temporal variation of the diatom abundance significantly (site*time: *F*(1,40) = 4.582, *p* = 0.038, Table 3). After two weeks the diatom abundance was more than ten times higher on samples in the pelagic zone (1001 ± 192) in comparison to the benthic zone (81 ± 42). A twofold difference between both habitats was observed at the second sampling time. There were no significant differences between the plastic types, however, after one month PE samples tended to have a smaller number of diatoms than biodegradable samples in both habitats (Fig 3).

**Table 3:** Diatom abundance in correlation to plastic type, site and exposure time.

	*SS* ^a^	*df* ^b^	*F* ^c^	*p* ^d^
plastic type	234.5	1	2.688	0.109
site	1027.9	1	11.784	0.001
time	1180.5	1	13.534	<0.001
plastic type*site	39.2	1	0.450	0.506
plastic type*time	32.0	1	0.366	0.548
site*time	399.6	1	4.581	0.038
plastic type*site*time	19.3	1	0.221	0.641
Residuals	3489.1	40		

^a^ sums of squares

^b^ degrees of freedom

^c^ *F*-value

^d^ probability value

**Fig 4:**
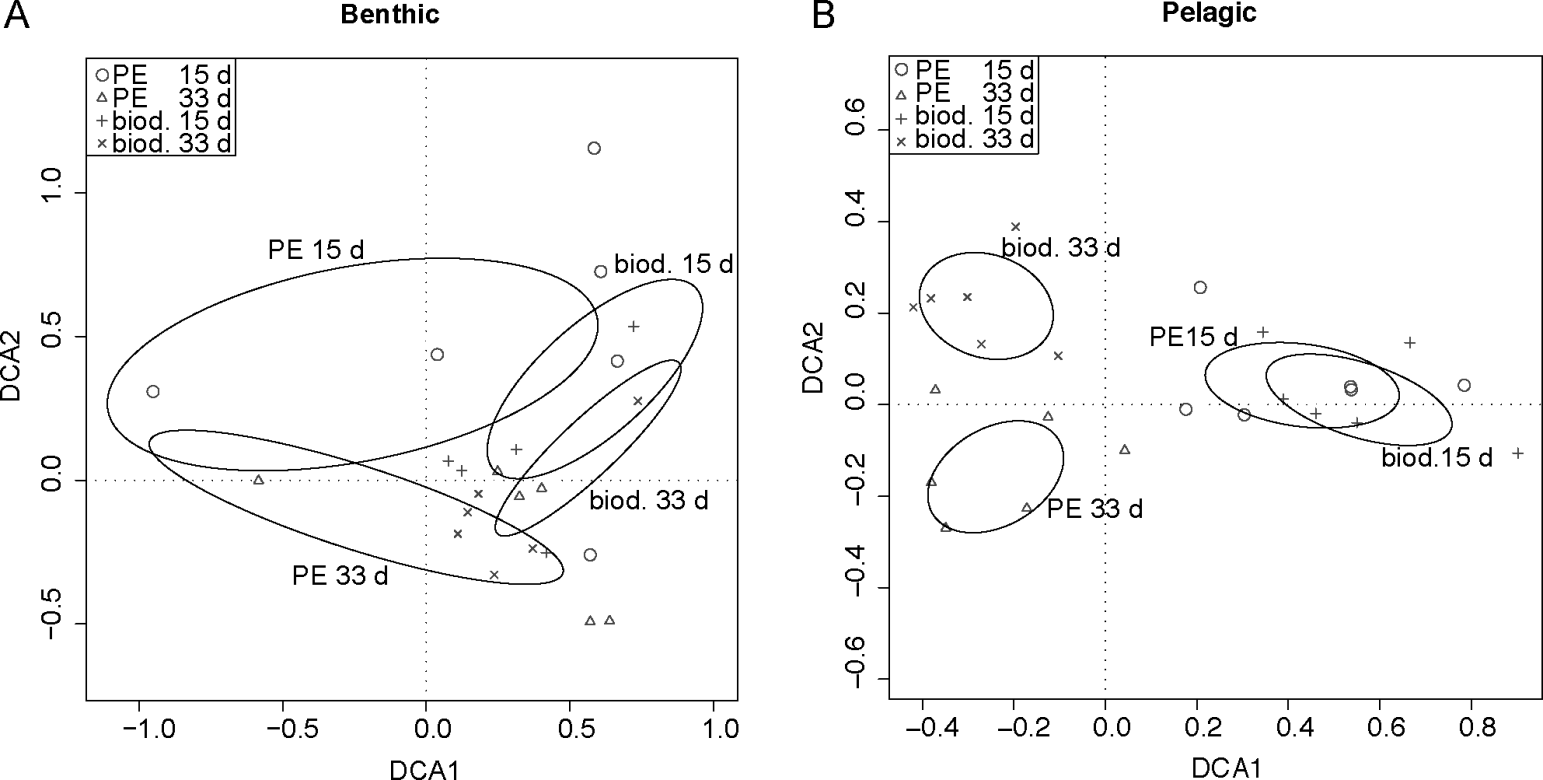
Detrended correspondence analysis (DCA) for the diatom community on PE and biodegradable plastic in the benthic (A) and in the pelagic zone (B). Diatoms were categorised by morphology. Each data point represents a plastic sample, the distance between the data points shows the similarity of the diatom composition found on the sample. The smaller the distance, the more similar the composition was between the samples. Different symbols were used to group the samples by plastic type and sampling time. Around the centroid of all data points for these groups an ellipse indicating the 95% confidence interval of their standard error was drawn. If these ellipses do not overlap the groups are assumed to be significantly different (P ≤ 0.05).

The diatom composition differed between the samples from the two sites (Fig 4). The two non-overlapping ellipses for the groups “biod. 33d” and “PE 33d” in Fig 4B show that after 33 days the diatom composition on pelagic samples was significantly different on biodegradable plastic from that on PE. For instance, the number of the diatoms of *Striatella* sp. was higher on biodegradable plastic in comparison to conventional plastic after 33 days. After 15 days these differences were not yet significant recognizable by the overlapping ellipses for the two plastic types at this time point. For the benthic samples, however, all ellipses overlapped showing that such differences between the plastic types did not exist.

### Oxygen production

**Fig 5:**
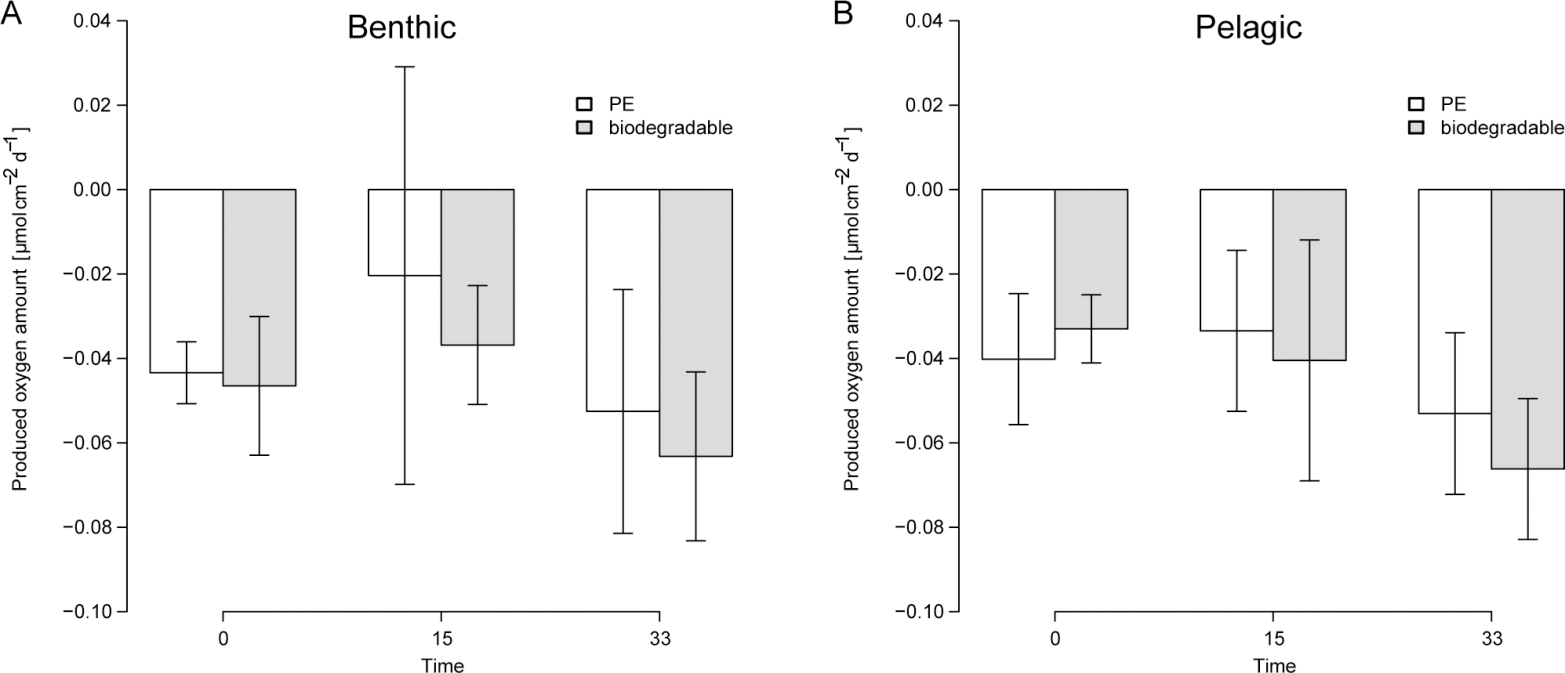
Oxygen production rate. Amount of oxygen (µmol) produced per day per square centimetre of plastic surface (white for PE, grey for biodegradable plastic) after 15 days and after 33 days of the experiment. Time 0 represents the polymer without biofilm. The error bars indicate the standard error. (A) Results of benthic samples (6 m water depth). (B) Results of pelagic samples (3 m water depth).

The samples showed negative values of oxygen production at all sampling times (Fig 5). Even unexposed samples had negative values. The high standard errors revealed large variability in the oxygen production during the early stage of fouling. The averaged values ranged from -0.02 to -0.07 μmol cm^-2^ d^-1^ (Fig 5). No statistical tests were performed due to the high standard deviation.

### Tensile properties

The results of the dynamometer are given in Table 4. The force needed to break samples (sigma) had values of 6.6 to 6.8 N at time 0 and showed a decreasing trend for all samples over time. The maximal elongation at break (epsilon) was between 410 and 499% at time 0 and displayed no clear trends during the experiment. No statistical tests were performed because of the high variation between the replicates, the low number of replicates and only relatively small differences between the treatments.

**Table 4:** Tensile properties (sigma and epsilon) for each treatment.

**Habitat**	**Plastic**	**Sigma (*N* ± *SE*** ^a^ **)**		
		0 days	15 days	33 days
Pelagic	PE	6.6 ± 0.8	6.1 ± 1.0	6.0 ± 0.3
Pelagic	biodeg.	6.8 ± 0.4	6.1 ± 0.3	6.1 ± 0.6
Benthic	PE	6.6 ± 0.8	5.6 ± 0.4	6.4 ± 1.0
Benthic	biodeg.	6.8 ± 0.4	6.2 ± 0.3	6.4 ± 0.2
		**Epsilon (% ± *SE*** ^a^ **)**		
		0 days	15 days	33 days
Pelagic	PE	499.0 ± 42.4	415.0 ± 40.1	472.8 ± 23.5
Pelagic	biodeg.	410.0 ± 54.9	288.0 ± 12.3	398.8 ± 68.9
Benthic	PE	499.0 ± 42.4	412.0 ± 34.9	464.5 ± 43.3
Benthic	biodeg.	410.0 ± 54.9	309.0 ± 29.6	332.0 ± 30.8

^a^ standard error

### SEM

**Fig 6:**
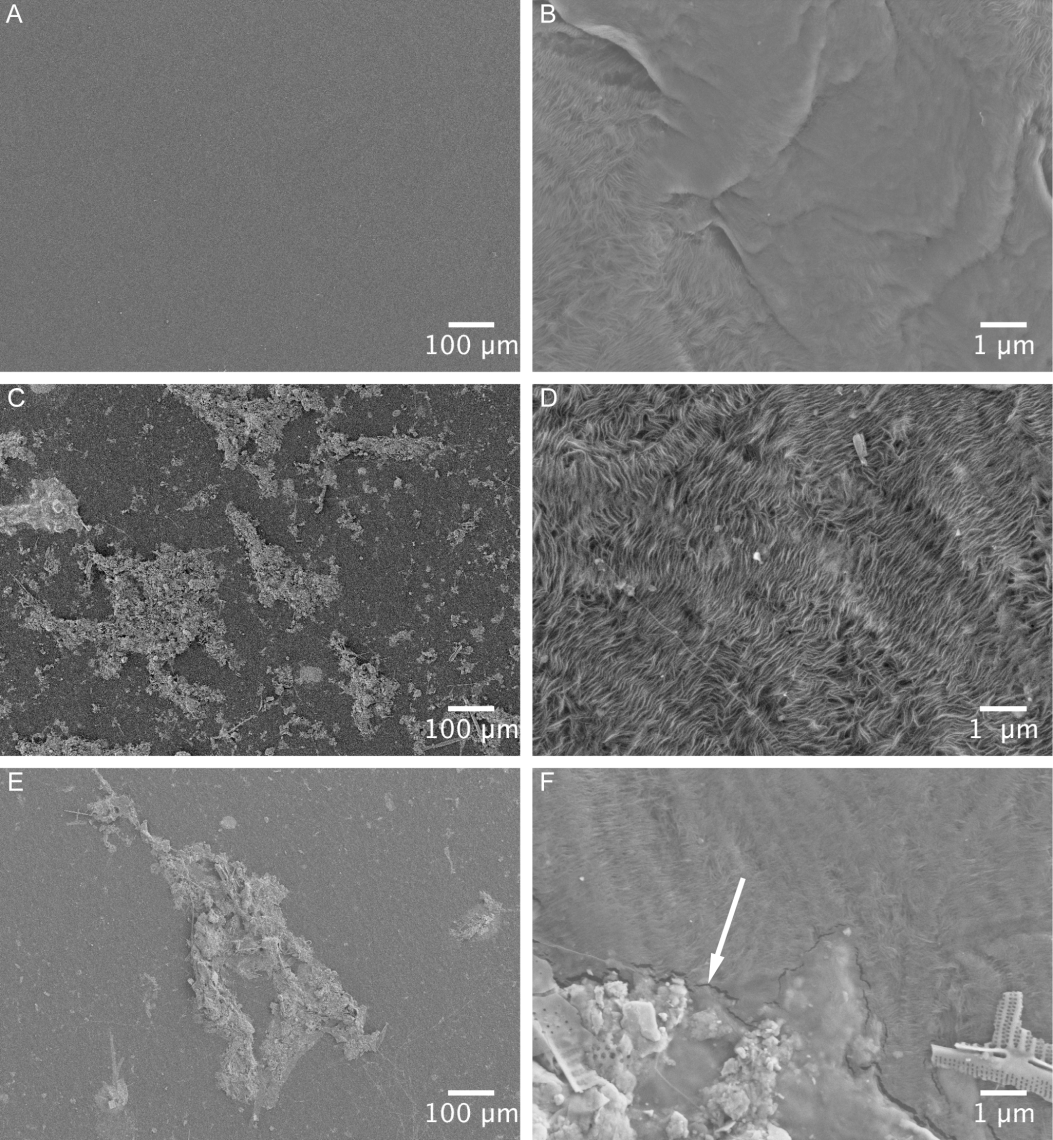
SEM pictures of the plastic surface of PE samples following removal of the biofilm. Each sample is displayed in two magnifications. (A) and (B) show untreated PE; (C) and (D) PE after 15 days; (E) and (F) PE after 33 days. Arrows in (F) mark fissures close to remains of the biofilm.

**Fig 7:**
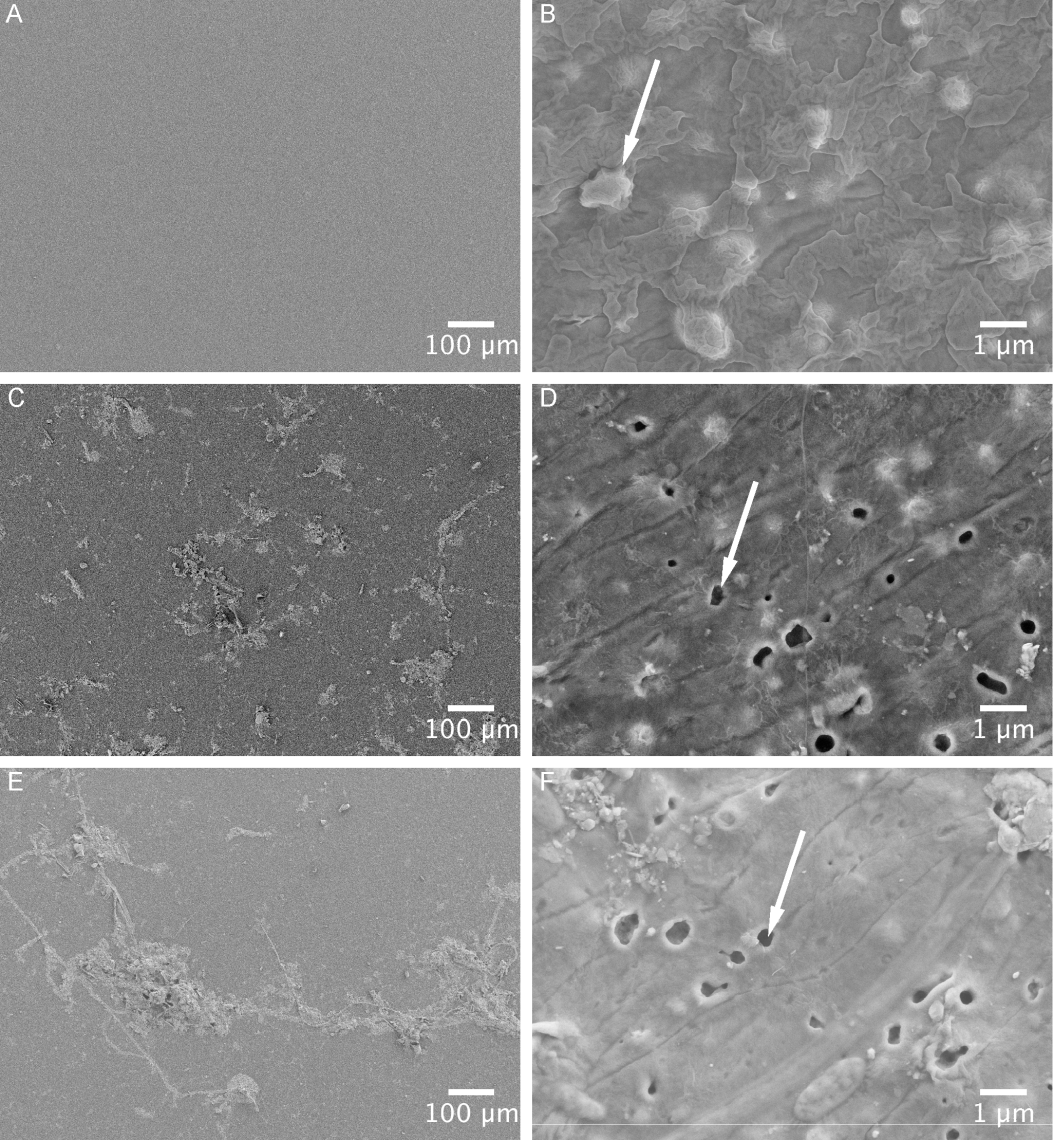
SEM pictures of the plastic surface of biodegradable plastic samples following removal of the biofilm. Each sample is displayed in two magnifications. (A) and (B) show untreated samples of biodegradable plastic; (C) and (D) of biodegradable plastic after 15 days; (E) and (F) of biodegradable plastic after 33 days. Arrows in (B) mark apparent starch particles and in (D) and (F) remaining holes.

Scanning Electron Microscopy analysis (Figs 6 and 7) displayed alterations in the surface properties of the biodegradable plastic within an exposure time of two weeks. On untreated biodegradable plastic apparent starch granules were visible (arrow in Fig 7B). After two and four weeks exposure time, small holes became visible in the smooth surface of biodegradable plastic samples from both habitats (arrows in Fig 7D and 7F). PE samples did not develop such holes within the study period, instead small fissures were observed (arrow in Fig 6F).

## Discussion

After 15 days of exposure to the marine environment a biofilm formed on the plastic surface of both plastic types and in both studied habitats. Similar observations were made for plastic that was exposed two meters below the water surface in the English Channel [25]. We conclude that a biofilm develops on plastic litter as soon as it is introduced to the marine environment, no matter to which habitat.

We assumed that microorganisms that feed on organic particles within the biodegradable plastic sample [20] create favourable conditions for the settlement of further microorganisms or provide food for higher trophic taxa and may thus increase the total biofilm amount on this plastic type. However, in this study the results show that during the first month the two plastic types had a similar total biofilm amount and diatom abundance.

Despite the occurrence of oxygen producing microorganisms like diatoms, the results of the oxygen measurements suggest that the overall oxygen balance is negative (Fig 5). We assume that this observation is mainly caused by oxidising of the polymers, a well-known process studied by polymer scientists [40–42]. For our data we cannot differentiate between this chemical polymer oxidising and biological processes like respiration and photosynthesis but we suggest that in the early stage of degradation and biofilm formation polymer oxidation is more dominant than biological processes.

The study period of 33 days did not reveal changes in the tensile properties of both plastic types. The high standard error of the measurement may be a result of a high variance between replicates caused by different polymer orientation in the plastic samples from the shopping bags. Plastic materials consist of a biaxial orientated polymer structure [43]. Depending on the pulling angle used during the dynamometer measurement in relation to the polymer structure the tensile strength parameters could differ. However, the exposure time of our study might be too low for changes in tensile properties. This is in concordance with other studies showing significant plastic degradation in the marine environment (measured by means of change in tensile strength, weight loss or surface area reduction) during long-lasting experiments performed for six months or up to one year [21,22,26]. SEM surveys can already elucidate early degradation processes in plastic [44], even when changes in tensile strength or mass loss are not yet detectable. Our SEM analysis indicated an initial degradation of the biodegradable plastic. The plastic surface showed holes after 15 and 33 days (Fig 7D and 7F), which might have developed due to dissolving or mineralisation of starch granules. Similar observations were made for a PE starch blend immersed in the Baltic Sea [45] and in the Bay of Bengal [40]. The degradation of the starch particles leads to an increase in plastic surface, which could enhance the degradation of the remaining polymer [20,45]. Considerable changes in the surface structure could not be observed for the PE plastic type. PE samples showed small fissures on the plastic surface particularly around remains of biofilm structures (Fig 6F). However, it cannot be excluded that surface tensions during the drying process of the samples caused these fissures.

In comparison to each other the surface structures of the two plastic types seem to be different (Figs 6 and 7). It is known that the surface texture is important for the community structure of a biofilm: for example, the diatom community differs between glass and fibreglass as substrates [46]; hence the plastic surface structure may affect the diatom community. The analysis of diatom communities (Fig 4) shows that after 15 days the community on both plastic types at the pelagic habitat was similar but differed after 33 days (although no statistical difference in the total diatom abundance could be revealed). A kind of specialisation seemed to have taken place probably due to the small-scale differences in the plastic surface structure and its physicochemical properties. On biodegradable plastic from the pelagic habitat more specimens of *Striatella* spec. were found after 33 days than after 15 days. This diatom species can attach to the substratum using a stalk [47], therefore we expect it to be highly dependent on the substratum surface properties, such as hydrophobicity. For samples from the benthic habitat this pattern was not observed. This might be due to a colonisation on these samples, which is disturbed by environmental events like wave action and lower light intensities. Furthermore we assume that microorganisms that feed on starch particles within biodegradable plastic may increase in abundance and thus are able to outcompete other organisms e.g. some diatom species.

Ecological implications of plastic debris on the benthic ecosystem are to date poorly examined. For example, plastic covering the sediment may inhibit gas exchange between the water column and the pore water within the sediment [48]. Furthermore, plastic debris may serve as a substrate favouring the establishment of some organisms and thus change the benthic community structure [49]. The surface of plastic provides a habitat for biofilms and in proceeding succession to other organisms. The scope of the biofilm amount on marine litter and its role in the ecosystem element cycle is not yet assessed.

Our results show that the community composition during the early colonisation depends on the habitat. Both the diatom abundance and their composition varied significantly between the two habitats. The diatom abundance was higher on floating plastic pieces. The habitats provide different physical, chemical and biological conditions. We conclude that re-suspended sand grains scratching along the plastic surface and a lower light intensity hindered the diatom succession in the benthic habitat. We assume that the differences between pelagic and benthic samples enlarge with increasing exposure time of the samples.

This study shows that plastic exposed to the marine environment is subject to biofilm formation within one month. In this time plastic type and habitat conditions influence the composition of biofilm, however not its total amount. The field study presented here confirms first signs of degradation of biodegradable plastic as has already been shown in laboratory tests. Additionally, our study shows that early degradation and biofilm formation are not affected by the habitat. It remains to be tested if this holds true during long-term experiments or if the degradation rate differs between habitats and how this affects the ecosystem.

## Supporting Information

S1 Dataset Raw data for biofilm amount measurements. (CSV)

S2 Dataset Raw data for diatom abundance and composition. (CSV)

S3 Dataset Raw data for oxygen measurements. (CSV)

S4 Dataset Raw data for tensile properties measurements. (CSV)
